# Graphene-Doped Ammonium Oxalate-Derived Carbon Aerogel with Controllable Structure for Synergistic Endothermic-Insulating Efficient Thermal Protection

**DOI:** 10.3390/gels12060535

**Published:** 2026-06-14

**Authors:** Zhengyang Lu, Guomin Ding, Qilin Mei, Borui Zheng, Kun Chen, Hong Wang, Xu Han, Jiayang Shao

**Affiliations:** School of Materials Science and Engineering, Wuhan University of Technology, 122 Luoshi Road, Wuhan 430070, China

**Keywords:** graphene-doped modification, superfine cooling materials, carbon aerogel, synergistic endothermic-insulating effect, thermal protection

## Abstract

High-performance thermal protection materials are urgently required in harsh thermal environments, such as hypersonic vehicles, the thermal runaway of energy batteries and high-temperature equipment. Conventional aerogels only exhibit passive thermal insulation and fail to resist instantaneous high-temperature attack. Herein, a cooling material of ammonium oxalate (AO) was introduced to achieve efficient, active endothermic protection. A cellular isolation effect induced by graphene nanosheets combined with anti-solvent crystallization was adopted to significantly decrease the size of AO crystals by over 93%. Based on superfine morphology and the constructed conduction network, the decomposition rate and heat absorption capacity of obtained graphene-doped AO powders (GdAPs) are improved by 41.2% and 30.4%, respectively. The mechanisms of morphology regulation and enhanced heat absorption are explored specifically in this study. Furthermore, GdAPs are embedded in phenolic resin to prepare thermal protection composite materials. Benefiting from their nearly complete thermal decomposition, GdAPs serve as a sacrificial template to generate discrete micropores in pyrolyzed resin. So, the as-prepared carbon aerogels (CAs) with a regulable microstructure exhibit an extremely low thermal conductivity of 0.056 W/(m·K), which is lower than those of reported CAs with the same density. Based on the above advantages, a synergistic endothermic-insulating thermal protection material is reported for the first time, and its heating rate is only 28.6% of that of commercial silica aerogel under identical high-temperature shock. Therefore, a new accessible strategy is demonstrated to provide high-efficiency thermal protection for resisting both abrupt and prolonged high temperature.

## 1. Introduction

In recent years, with the improved safety requirements for aerospace hypersonic vehicles, high-energy-density batteries and extreme industrial thermal equipment, thermal protection materials (TPMs) against instantaneous thermal attack and prolonged high-temperature exposure have attracted significant attention [1,2,3]. As a type of porous material with low thermal conductivity, aerogels show notable advantages in reducing solid heat conduction and limiting thermal convection, so they are regarded as ideal candidates for TPMs [4,5]. In particular, carbon aerogels (CAs) with low density and outstanding heat resistance, which can provide thermal protection under extreme heat environments, have become a research hotspot [6,7]. Nevertheless, the natural high thermal conductivity of carbon materials conflicts with the insulating demand of CAs. Therefore, rational microstructure design is critically essential for improving the thermal insulation of CAs [8]. However, CAs are predominantly derived from the pyrolysis of polymer precursors nowadays, which generally produces random networks. This poor structural tunability restricts the further development of CAs in advanced thermal protection systems [9,10].

In addition, most conventional aerogels only exhibit passive thermal insulation function, resulting in weak protective performance against instantaneous high-temperature shock. By contrast, active thermal protection strategies, including transpiration cooling [11], phase change energy absorption [12] and directed convective heat removal [13], exhibit good adaptability for abrupt thermal attack. Therefore, an active–passive combined thermal protection strategy has emerged as a prominent research focus in recent years [14,15]. However, the reported active thermal protection methods mainly depending on the embedding of micro-channel cooling structures, phase change microcapsules or active heat dissipation components in TPMs suffer from large weight, high cost, complex processes, and poor structural stability.

In addition, heat absorption efficiency for active thermal protection is a key factor dominating overall protective performance. Compared with physical phase change heat absorption, cooling materials capable of endothermic chemical decomposition possess much higher heat absorption capacity [16,17,18]. In contrast to traditional endothermic compounds such as Al(OH)_3_ and Mg(OH)_2_, ammonium oxalate (AO) displays irreplaceable competitiveness owing to its ultra-high specific endothermic decomposition enthalpy of about 1500 J/g [19,20]. Additionally, the gaseous products of decomposed AO, including N_2_, CO_2_ and H_2_O, are all environmentally benign and can be completely discharged, rendering AO highly suitable for cleanliness and lightweight TPM [21,22,23]. Nevertheless, commercial AO has a large particle size and low thermal conductivity, resulting in inferior decomposition kinetics and a slow decomposition rate. Therefore, it is difficult to meet strict requirements for rapid isolation against high-temperature shock [24,25].

Herein, to improve the thermal response speed and endothermic performance of AO, a graphene doping modification combined with anti-solvent crystallization technique was adopted to significantly decrease the size of AO crystals and fabricate superfine AO powders. Based on the morphology regulation and thermal conductivity of the constructed graphene network, the decomposition rate and heat absorption of AO were enhanced by 41.2% and 30.4%, respectively. Moreover, by means of superfine AO powders as sacrificial templating embedded in phenolic resin (PR), the microstructure of CAs that resulted from the pyrolysis of resin could be controlled, and an extreme low thermal conductivity of CAs was achieved. Based on the strong heat absorption of AO cooling materials and efficient thermal insulation of CAs, a new endothermic-insulating coupled TPM was designed and prepared in this study, which shows superior resistance to instantaneous high-temperature shock and excellent long-term heat insulation.

## 2. Results and Discussion

### 2.1. Morphology Control for AO via Anti-Solvent Crystallization

**Figure 1:**
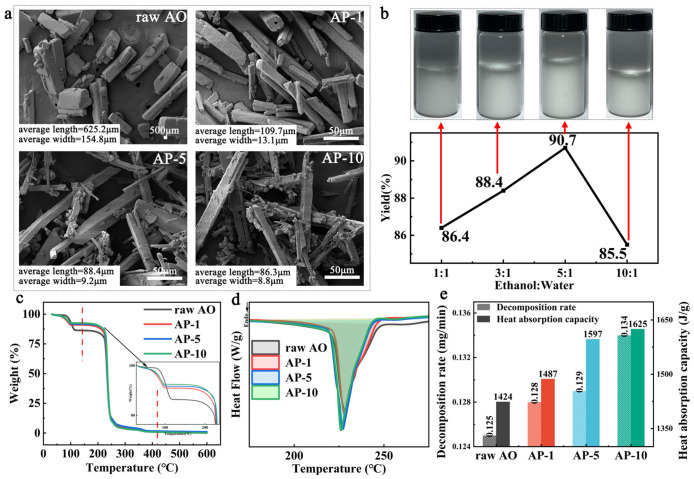
(**a**) SEM images of AOs obtained from different anti-solvent systems. (**b**) Yield of recrystallized AOs in different anti-solvent systems. (**c**,**d**) TGA and DSC curves of different AOs. (**e**) Heat absorption capacity and decomposition rate of AOs calculated based on TGA and DSC curves.

The small size of cooling materials is beneficial to improving heat absorption capacity because of the large specific surface area for enhancing the reactivity of chemical reactions [26]. However, commercial AO usually has a large size, so it exhibits both low heat absorption and slow decomposition rates. To decrease the size of AO crystals, an anti-solvent of ethanol was added into the AO aqueous solution to induce recrystallization and regulate its morphology. Based on the volume ratio of ethanol to water, which is 1:1, 3:1, 5:1 and 10:1, the obtained AO powders were designated as AP-1, AP-3, AP-5, and AP-10, respectively. The influence of ethanol content on the morphology of AO crystals was first investigated. The results indicate that compared to raw AO (average length of 625.2 μm, average width of 154.8 μm), the AP-1 crystals have an obviously smaller size with an average length of 109.7 μm (a reduction of 82.45%) and an average width of 13.1 μm (a reduction of 91.56%), as shown in Figure 1a. As the ethanol content increases, the average length and width of AO crystals further decreased to 95.9 μm and 9.4 μm for AP-3, 88.4 μm and 9.2 μm for AP-5, and 86.3 μm and 8.8 μm for AP-10, respectively (Figure 1a). Based on the particle size distribution, the obtained AP-3 (Figure S1), AP-5, and AP-10 meet the standard for superfine powders (particle width < 10 μm) [27]. The above changes are mainly attributed to the drastic decrease in the solubility of AO upon the introduction of ethanol as the anti-solvent, which generates a high supersaturation level, promoting nucleation while suppressing crystal growth [28,29]. In addition, all crystals exhibit a typical cuboid structure, indicating that they belong to the orthorhombic crystal system. This consistent crystal structure was confirmed by the same XRD patterns in Figure S2, which shows that the anti-solvent crystallization process only decreases the size of AOs and does not change their lattice structure. Moreover, the yield of AO recrystallized from different solvent systems was also measured and calculated. The results indicate that as the ethanol contents increase, the yield of AOs first rises, reaching a maximum of 90.7% for AP-5, and then decreases (Figure 1b). This can be attributed to the decreasing solubility of AO in the anti-solvent system at the initial stage, thereby triggering increased crystal precipitation. But once excessive ethanol is added, even if the solubility of AO in the solution decreases further, the increased amount of solvent also leads to a higher dissolved content, resulting in a decrease in yield.

Furthermore, the decomposition behavior of the above AO crystals was investigated. Their TGA curves reveal that AO exhibits two-stage decomposition (Figure 1c). The first stage of weight loss occurs around 70 °C, primarily involving the removal of water molecules. The results show that the weight loss rate of AP-1 in this stage was measured at 8.2%, which decreases by 39.7% compared to that of raw AO (13.6%). And then as ethanol content increases, this rate continues to decrease. This reducing water component in recrystallized AOs is due to the decreased water content in the anti-solvent system, consequently lowering the levels of both bound and adsorbed water in the obtained crystals. This inherent low water content favors a higher AO molecular content in crystals, which will translate into a direct benefit for improved specific heat capacity. The decomposition of AO molecules occurs in the second stage, with an initial decomposition temperature of about 228 °C, corresponding to the main endothermic process. The decomposition rates of the prepared AOs show a positive correlation with increasing anti-solvent content. Compared to raw AO (decomposition rate of 0.125 mg/min), the decomposition rates increase to 0.128 mg/min for AP-1, 0.129 mg/min for AP-5 and 0.134 mg/min for AP-10, corresponding to increases of 2.48%, 2.8% and 6.95%, respectively. This is primarily due to the reduction in particle size, which increases the surface area exposed to heat, thereby accelerating the decomposition process. However, it must be acknowledged that the effect of merely reducing the particle size of AO crystals on the decomposition rate is indeed relatively small. Additionally, the heat absorption capacity of AOs was calculated by integrating the DSC curve, as shown in Figure 1d. The results show that the heat absorption capacity increases monotonically from raw AO to AP-10. In particular, the absorption capacity of AP-5 (1597 J/g) and AP-10 (1625 J/g) is over 12% higher than that of raw AO (1424 J/g), as shown in Figure 1e. This is attributed to the fewer defects and more complete decomposition of AO with a small size. The similar effect of particle size on heat absorption for other materials has been verified in reported studies [30,31,32,33]. Finally, all AOs can be completely decomposed without any residues, as shown in TGA curves. To sum up, considering that it had the highest yield, a nearly saturated decomposition rate and endothermic capacity, the anti-solvent system with a volume ratio of ethanol to water of 5:1 for preparing AP-5 was adopted for subsequent studies.

### 2.2. Superfine Graphene-Doped AO Powders (GdAPs)

**Figure 2:**
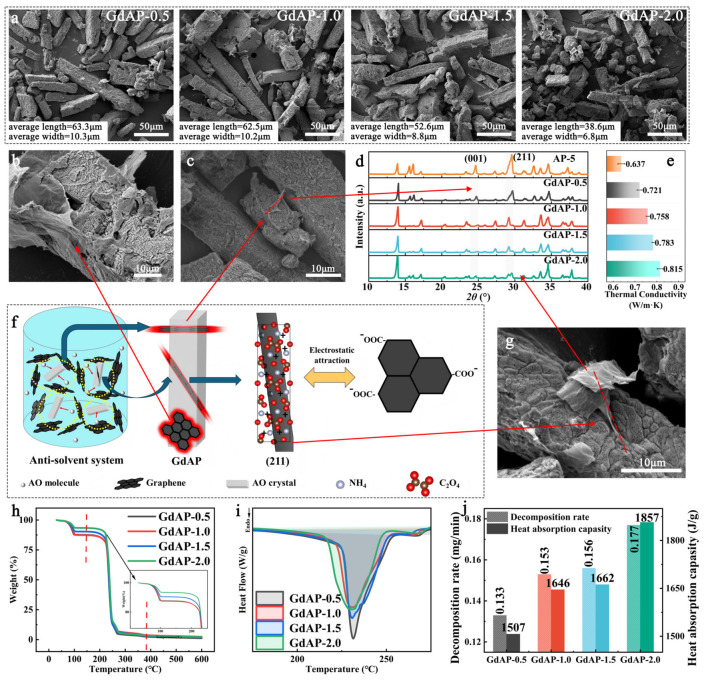
(**a**) SEM images of GdAPs with different graphene contents. (**b**) Graphene coated on AO surface. (**c**) Graphene vertically intercalated in AOs. (**d**,**e**) XRD pattern and thermal conductivity of different GdAPs. (**f**) Schematic illustration of graphene-doped AO mechanism. (**g**) Graphene obliquely intercalated in AOs. (**h**,**i**) TGA curves and DSC curves of different GdAPs. (**j**) Decomposition rates and heat absorption of different GdAPs.

As shown in Figure 1a, although the width of AOs is decreased by means of anti-solvent crystallization, the crystals do not become obviously short, as a large length of about 90 μm is still shown for AP-5. To further enhance endothermic capacity, particularly to accelerate the endothermic rate, the length of AOs should be decreased, and a novel strategy to promote decomposition needs to be developed. In this study, the graphene obtained from reducing GO with immense specific surface area and abundant crystallization sites [34,35,36,37] was introduced in the anti-solvent crystallization process, which is expected to miniaturize the axial dimension of AO crystals. Combining chemical reduction using ascorbic acid, the graphene nanosheets were prepared during anti-solvent crystallization by the one-step method. Based on the graphene weight ratios to AO powder of 0.5 wt%, 1.0 wt%, 1.5 wt%, and 2.0 wt%, the obtained composite powders were named GdAP-0.5, GdAP-1.0, GdAP-1.5 and GdAP-2.0 respectively. The influence of graphene content on AO morphology is characterized in Figure 2a. The results indicate that in the presence of only a small amount of graphene, the length of GdAP-0.5 crystals decreases obviously to 63.3 μm. As graphene content increases, GdAPs are further shortened, and GdAP-2.0 possesses the smallest length, with a 56.3% decrease relative to the graphene-free crystals of AP-5. It should be noted that when a small amount of graphene is present, GdAP-0.5 crystals show a larger width than AP-5 crystals. When the graphene content further increases, the average width of GdAPs also decreases gradually. Finally, GdAP-2.0 with the highest graphene content in this study shows an average length of 38.6 μm and average width of 6.8 μm, which decrease by 93.8% and 95.6% compared to those of raw AO. In addition, all GdAPs still show a regular cuboid shape, indicating that the introduction of graphene does not alter the crystal structure of AOs.

The above regulating mechanism carried out by graphene for AO morphology is illustrated in a schematic diagram in Figure 2f. We consider that the dispersed graphene nanosheets with large surface area will cause a cellular isolation effect, which divides the solution system into numerous independent spaces. This spatial confinement will hinder crystal growth, which accounts for the significant reduction in length (dashed boxes in Figure 2f). In this case, the AO molecules will inevitably accumulate in the radial direction, thus slightly increasing the width of GdAPs with a low graphene content. Then with a rise in graphene content, the spaces divided by the nanosheets become smaller, leading to a decrease in AO molecules in encapsulated cells. Therefore, both the length and width of the obtained GdAPs decrease continually.

Moreover, different binding modes between graphene and AO crystals were identified through detailed observation. The first mode involves graphene covering the surface of the AO crystals, as shown in Figure 2b. This is easy to understand because of the deposition of free graphene in the solution onto the crystal surface during the filtration process. In addition, we find that some graphene nanosheets are inserted into the AO crystals, mainly including two types: vertical insertion (Figure 2c) and oblique insertion (Figure 2g). The above insertion states were also verified by XRD spectra. As shown in Figure 2d, compared to pure AP-5, GdAPs exhibit gradually weakening diffraction peaks at 2θ = 24.6° and 2θ = 29.5° with an increase in graphene content, which correspond to the (001) and (211) crystal planes of the AO crystal based on Powder Diffraction File. These are exactly the crystal planes occupied by vertically inserted graphene and obliquely inserted graphene, respectively. In our opinion, vertical intercalation results from graphene inhibiting AO crystal growth along the axial direction, leading to the nanosheets encapsulated internally (Figure 2f). Additionally, the AO unit cell is a sandwich layer stacked perpendicularly along the axial direction via weak Van der Waals forces and hydrogen bonds [38]. Graphene intercalates along the (001) crystal plane with the weakest bonding forces, presenting a low energy barrier. Moreover, the formation of the oblique insertion layer is attributed to the affinity of the (211) surface with the NH_4_^+^ ions in AO, thereby promoting stable electrostatic attraction with negative graphene nanosheets because of the ionization of residual carboxyl groups, as shown in Figure 2f.

Next, the decomposition characteristics and heat absorption capacity of GdAPs were characterized (Figure 2h,i). The experimental results indicate that the introduction of graphene did not alter the decomposition behavior of AO, which still contains two obvious decomposable stages. With the introduction of graphene, the weight loss rate in the first stage decreases progressively. GdAP-2 exhibits a first-segment weight loss rate of only 6.5%, a 20.4% reduction relative to pristine AP-5. This indicates that introducing graphene will further reduce the water content in AO. In our opinion, this is primarily attributed to the strong hydrophobicity of graphene [39,40], which will repel water molecules from absorbing on AO crystals during crystallization. More importantly, TGA indicates that as the graphene content increases, the decomposition rate in the second stage increases significantly from 0.133 mg/min for GdAP-0.5 to 0.177 mg/min for GdAP-2.0 (Figure 2j), which represents a 37.8% increase compared to AP-5 and a 41.2% increase compared to raw AO. We suppose that such high improvements are primarily attributed to two factors. Firstly, the reduced particle size increases the specific surface area of GdAPs further, which provides a greater active surface for heat exposure, accelerating surface decomposition. Secondly, because of excellent conductivity, the graphene nanosheets covered on the surface and intercalated in crystals will form efficient thermal conduction pathways. This facilitates rapid heat transfer into the crystal interior and additionally enables bulk decomposition (Figure 2f). The improved conductivity enabled by doping graphene is verified by an increase in thermal conductivity values in Figure 2e. In addition, we tested and calculated the heat absorption capacity of GdAPs. The results showed that it gradually increased from 1507 J/g for GdAP-0.5 to 1858 J/g for GdAP-2.0, representing a 16.3% increase compared to AP-5 and a 30.4% increase compared to raw AO. This is primarily due to the substantial interfacial area introduced by graphene nanosheets, which requires additional heat absorption during the heating process to accommodate interfacial stress relaxation and the rearrangement of constrained molecular configurations [41]. Meanwhile, the thermal conduction network formed by graphene in AO crystals improves the efficiency of heat delivery. This allows crystals to undergo more complete decomposition, thereby releasing the maximum endothermic potential of AO. In addition, due to the extremely low graphene content and carbonization at high temperatures, the residue of all GdAPs is less than 1%, indicating that this composite powder is nearly entirely consumed after decomposition.

### 2.3. Improved Strength and Thermal Insulation for Composite Precursor and CAs

**Figure 3:**
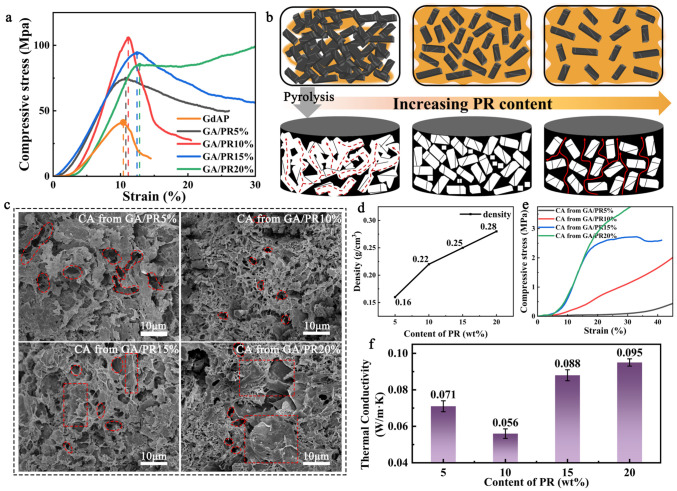
(**a**) Compressive strength of GA/PRs. (**b**) Structure diagrams of GA/PRs and corresponding CAs. (**c**) SEM images of CAs from GA/PRs. (**d**,**e**) Density and compressive curves of CAs from GA/PRs. (**f**) Thermal conductivity of CAs from GA/PRs.

To achieve active–passive combined thermal protection, this study expects to incorporate the high heat absorption property of GdAPs and the thermal insulation capability of CAs. Therefore, we mix superfine GdAP-2.0 and phenolic resin (PR) to prepare a corresponding composite precursor. When the precursor is exposed to high temperatures, the inner GdAPs will decompose rapidly, thereby absorbing a large amount of heat. In addition, the closed micropores left by the completely decomposed superfine powder and the carbon skeleton from the pyrolyzed resin can form aerogels, leading to long-term thermal insulation performance. This study selected PR as the matrix because of its excellent ability to prepare the stable carbon skeleton [42,43]. According to the different mass percentages of PR to GdAPs, the obtained GdAP/PR composite materials are designated as GA/PR5%, GA/PR10%, GA/PR15% and GA/PR20%. The effect of resin content on the mechanical performance of GA/PRs was studied firstly. The results indicate that the compressed strength of the pure GdAP briquette is relatively low, measuring only 41 MPa. As the resin content increases, the compressive strength of GA/PRs increases gradually from 75 MPa for GA/PR5% to 106 MPa for GA/PR10% (Figure 3a), representing a 157.9% improvement over the GdAP briquette. This high strength ensures the practicality of this composite material. However, with a further increase in PR content, the compressive strength of GA/PRs begins to decrease, dropping to 85.5 MPa for GA/PR20%. This change can be explained by the mechanism schematized in Figure 3b. For the pure GdAP briquette, there are many voids between powder particles, resulting in a loose structure and weak strength. As resin is added, it progressively fills these voids, bonding the dispersed particles into a whole. This state facilitates the formation of a continuous stress transfer network between GdAPs, significantly enhancing the mechanical properties of the composite. According to the variation in strength, we hypothesize when the PR content increases to about 10 wt%, the resin can fully enclose GdAP particles, forming a compact and intact composite structure. In this state, the bonding action and stress transfer capability of resin achieve optimal performance, leading to the maximum strength. When the resin content is excessive, the GdAPs are widely separated from each other by the thick resin layer. This hinders load transfer among particles and makes the performance of the composite close to that of the PR with lower strength compared to inorganic AO crystals. The above theory is also verified by the increasing fracture strain of GA/PRs in Figure 3a, which originates from the viscoelastic characteristics of the cured resin. Subsequently, the obtained GA/PRs were pyrolyzed at 950 °C (using an alcohol burner, Zhengzhou Xingyao Laboratory Equipment Co., Ltd., Zhengzhou, China) to prepare the corresponding CAs. As the resin content increases, the density of the obtained CAs monotonically increases from 0.16 g/cm^3^ to 0.28 g/cm^3^ (Figure 3d). This agrees with the trend that as resin content increases, the skeletal structure of the aerogels gradually increases. Therefore, their load-bearing capacity also improves accordingly (as shown in Figure 3e). It is noteworthy that aerogels formed at a low resin content do not exhibit typical failure points. This is attributed to their relatively loose structure, which will be continuously compacted under external force.

Then, the microstructure of the aerogels was characterized by SEM (Figure 3c). The results indicate that at a low resin content, the obtained aerogel exhibits an extremely irregular pore distribution, forming numerous interconnected holes exceeding 10 μm in diameter. At this point, the pore walls are thin and appear fragmented. When the PR content increases to 10 wt%, the diameter of micropores in corresponding CA decreases obviously to about 4 μm, which is approximately equal to the original geometric features of GdAPs. This indicates that the GdAP particles are well isolated by the PR, resulting in the formation of numerous discrete, uniform micropores, which presents a honeycomb-like porous framework (as marked by the dashed circles in Figure 3c). Under this condition, the micropore walls exhibit moderate thickness and smooth surfaces. This phenomenon is consistent with the above strength analysis for GA/PR10%. Then, as the resin content further increases, wall thickness increases significantly. Even when the resin content reaches 20 wt%, the obtained aerogel shows extensive and continuous wall structures, and micropores are buried within the dense bulk framework (as marked by the dashed boxes in Figure 3c). The difference in the microstructure of aerogels will directly influence their thermal insulation performance. The results indicate that as the resin precursor content increases, the thermal conductivity of aerogels first decreases and then increases (Figure 3f). This phenomenon stems from the coupling effect between the aerogel skeleton and micropores in different CAs. As shown in Figure 3b, when the PR content is low, the interconnected micropores in the carbon aerogel skeleton will enhance thermal convection and lead to high thermal conductivity. Then as PR content increases, the skeleton becomes continuous, which will isolate the formed micropores into independent spaces. In this case, discrete micropores can significantly suppress thermal convection, so thermal conductivity decreases. However, when the PR content is excessively high, the obtained CAs have a thick carbonized gel skeleton. Due to the good conductivity of carbon materials, solid-state heat transfer is enhanced, causing a subsequent rise in thermal conductivity. Finally, the obtained aerogel from GA/PR10% with a well-organized microstructure exhibits the lowest thermal conductivity of ~0.056 W/(m·K). Such a low thermal conductivity has not been reached in reported carbon-based aerogels with similar density, whose values typically range from 0.07 to 0.11 W/(m·K)) [44,45,46,47,48,49,50,51,52,53,54]. This is very beneficial to long-term thermal insulation for TPMs under high temperature. Therefore, the composite with a resin content of 10% is adopted in later studies. In addition, the decomposition rate and heat absorption capacity of GA/PR10% were also characterized, which were 0.173 mg/min and 1746 J/g, respectively (Figure S3). These slight reductions in decomposition speed and endothermic properties are due to the introduced resin, causing a decrease in the relative proportion of GdAPs in the composite material.

### 2.4. Synergistic Endothermic-Insulating Thermal Protection

**Figure 4:**
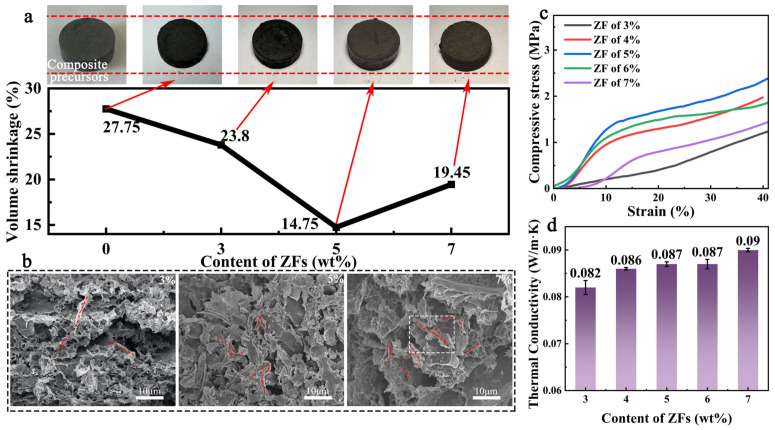
(**a**) Effect of ZF content on volume shrinkage. (**b**–**d**) SEM images, compressive curves and thermal conductivity of different CAs, respectively.

In the above studies, a significant volume shrinkage of the composite was observed during the thermal decomposition process (Figure S4). For GA/PR10%, the volume shrinkage rate reaches 27.75%. To address this issue, heat-resistant ZFs were introduced to provide enhanced structural support. The results indicate that ZFs play a crucial role in regulating dimensional stability for GA/PRs. When the ZF content reaches 5 wt%, the volume shrinkage rate drops to a minimum of 14.75%, representing a 46.8% decrease compared to the composite without ZFs (Figure 4a). However, as the ZF content increases further, the volume shrinkage rate of the aerogel instead increases. This phenomenon can be explained based on changes in the microstructure of aerogels. As shown in Figure 4b, when the ZF content is low, the fibers in the aerogel skeleton exhibit distinct sparseness, which are embedded in the resin skeleton, providing necessary support but failing to form an effective continuous support framework. With an increase in ZF content, the fibers begin to overlap each other and bond via pyrolytic carbon to form a three-dimensional rigid framework. This can effectively resist shrinkage stress and reduce the volume shrinkage of the aerogel. However, when ZF content is excessively high, the interfaces become very rich, which results in inadequate bonding between the fibers due to the relatively insufficient pyrolytic carbon. This results in part fibers in an exposed or suspended state, as shown in the dashed rectangles of Figure 4b, leading to a decrease in structural stability.

Benefiting from its enhanced microstructure, the CAs containing 5 wt% ZFs also exhibit the best mechanical properties, with a compressive strength reaching 1.27 MPa and an average modulus of 5.82 MPa (Figure 4c). Compared to the aerogel without fiber addition (compressive strength of 0.16 MPa under same strain), this represents an over 7 times improvement in compressive strength. In addition, the thermal conductivity of aerogels with different ZF contents was also characterized (Figure 4d). The results showed that their thermal conductivity slightly increases to the range of 0.08–0.09 W/(m·K). This phenomenon is attributed to the higher thermal conductivity of ZFs (2–3 W/(m·K)) than that of the carbon skeleton (0.1 W/(m·K)) [7,48,50]. Nevertheless, the composite aerogel still exhibits low thermal conductivity, enabling effective thermal insulation in practical applications. Based on their low volume shrinkage, excellent mechanical properties, and relatively good thermal insulation performance, 5 wt% ZFs are added to prepare composite materials with integrated heat absorption and insulation functions in another study. In addition, the prepared composite shows a relatively low density of 1.3 g/cm^3^, and its decomposition rate and heat absorption capacity are 0.172 mg/min and 1767 J/g, respectively (Figure S5), which still shows its improved decomposition and endothermic capacity. Furthermore, the corresponding aerogel has a porosity of 88.67% and a specific surface area of 259.03 m^2^/g.

**Figure 5:**
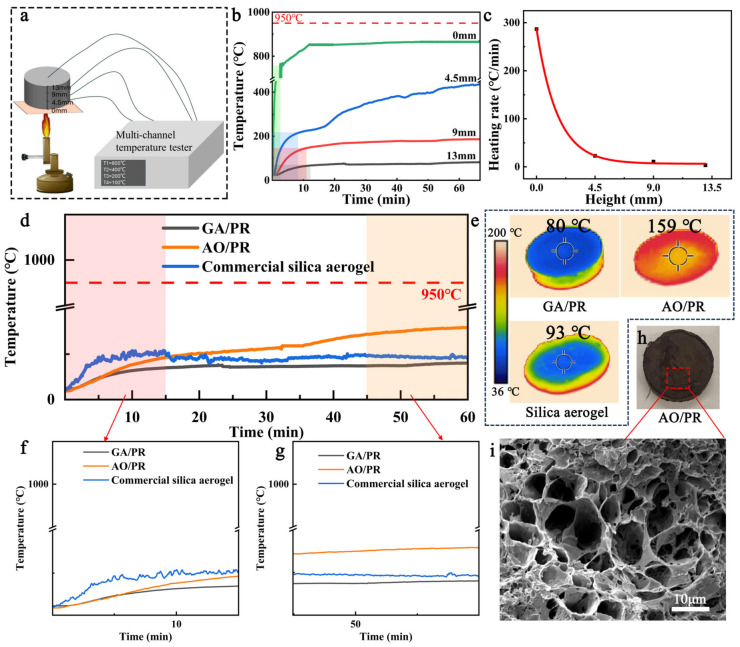
(**a**) Schematic diagram of simulation experiment. (**b**) Temperature curves at different height points. (**c**) Rate of temperature increase at different height points. (**d**) Surface temperature curves for different TPMs. (**e**) Infrared thermal images of different TPMs. (**f**,**g**) Partial surface temperature curves for different TPMs. (**h**,**i**) Photograph and microstructure of AO/PR aerogel.

To comprehensively evaluate the thermal protection performance of this composite material, a simulation experiment was designed by placing our composite (13 mm thick) on an alcohol burner (about 950 °C), and thermocouple sensors and a multi-channel temperature tester were employed to record real-time temperatures in specimens from the bottom to the top surface (Figure 5a). The results indicate that the temperature rise curve can be divided into heating and stable phases (Figure 5b). Based on heating behavior, the heating rates at different heights are calculated. It is evident that the heating rate decreases exponentially with increasing height, falling drastically from 286.8 °C/min at the bottom surface to 3.4 °C/min at the top surface, which indicates robust resistance against instantaneous high-temperature attack (Figure 5c). We suppose that such a significant change mainly originates from two aspects. Firstly, the decomposition of GdAPs will absorb a large amount of heat, resulting in less heat being transferred along the thickness direction. Secondly, the CAs formed by decomposed GdAPs and pyrolyzed resin will insulate heat transfer, leading to a further decrease in heat transport. Moreover, even after GdAPs are completely decomposed, benefiting from the good thermal insulation performance of formed CAs, the stable temperature decreases sequentially from 864 °C at the bottom surface to 80 °C at the top surface (Figure 5b). Based on the following formula, thermal insulation performance was evaluated by defining the temperature decay ratio (η) [55]:

(1) $$\eta =\frac{T_{1}-T_{2}}{T_{1}}\times 100\%$$

where *T*_1_ and *T*_2_ are the heat source temperature and surface temperature of the thermal protection material, respectively. The temperature decay ratio of our composite reaches 92%, validating an effective thermal insulation property.

To investigate the effect of the superfine morphology of AO crystals on thermal protection performance, we mixed raw AO, PR and ZFs at the same ratio to prepare comparative AO/PR samples with the same thickness as GA/PR. The variation in surface temperature under identical thermal attack was recorded. As shown in Figure 5d, the results indicate that the heating rate of the AO/PR surface is 4.8 °C/min, which is 1.4 times larger than that of GA/PR (Figure 5d,f). This is because that compared with superfine GdAP, raw AO exhibits a lower decomposition rate and weaker heat absorption capacity, as stated previously, resulting in a slow response and relatively weak active thermal protection. In particular, the final surface temperature of AO/PR reached as high as 159 °C, nearly twice as high as that of GA/PR, suggesting inferior passive thermal insulation performance (Figure 5e,g). This can be explained by the microstructure difference in corresponding CAs. As shown in Figure 5i, many large pores of about 15 μm exist in CA derived from pyrolyzed AO/PR because of its large size of raw AO. This will induce pronounced thermal convection and thus leads to unsatisfactory thermal insulation behavior. The thermal conductivity of this CA is measured and found to be up to 0.169 W/(m·K), which is far higher than that of our CAs. In addition, the existence of large-sized pores also leads to inferior structural stability, accompanied by distinct cracking behavior in this aerogel during the thermal pyrolysis process (Figure 5h).

Moreover, the change in surface temperature on a common silica aerogel (Figure S6, thermal conductivity of 0.027 W/(m·K)) with the same thickness as the above composite was also measured under the same thermal shock. The results indicate that the silica aerogel undergoes a rapid temperature rise with a heating rate of 11.9 °C/min, which is 3.5 times higher than that of GA/PR (Figure 5d,f). This demonstrates that active thermal protection derived from the endothermic decomposition of superfine GdAPs exhibits far superior resistance to transient high temperatures compared with silica aerogel. Moreover, due to higher thermal stability of CA than silica aerogel, the final stable surface temperatures of our CA are lower than those of silica aerogel, indicating better long-term thermal insulation capability (Figure 5e,g). In addition, although the endothermic component is exhausted, the formed CA can still continuously supply repeated passive thermal protection. It was found that after 10 thermal shocks, the heating rate of residual CA is 9.6 °C/min, still exhibiting good thermal insulation performance (Figure S7). Further tests at 1300 °C prove that ammonium oxalate decomposition products endow the aerogel with a well-preserved structure (Figure S8) and stable thermal conductivity of 0.088 W/(m·K) under extreme thermal shock. In summary, the GA/PR prepared in this study can provide timely active thermal protection and long-term passive thermal protection, which offers excellent comprehensive thermal protection for extreme high-temperature environments.

## 3. Conclusions

In conclusion, by anti-solvent crystallization and graphene-doped modification, the size of cooling material AO was decreased dramatically, and superfine GdAP crystals were prepared. The effects and mechanisms of the above two techniques on morphology and endothermic behavior were explored in detail. Based on the size regulation and conductivity of the graphene network, the decomposition rate and heat absorption capacity of GdAP were improved by 41.2% and 30.4%, respectively. Moreover, the obtained GdAP decomposed almost completely under high-temperature conditions. Therefore, we used it as a sacrificial template to introduce discrete micropores in pyrolytic CAs, so thermal insulation performance was improved significantly, but it was lower than that of reported CAs with the same density. Finally, a synergistic endothermic-insulating TPM combining a cooling material and CA was achieved for the first time. Because of the enhanced heat absorption of GdAP, the prepared composite TPM shows a much quicker thermal protective response than conventional aerogels. Moreover, it also possesses better long-term thermal insulation capability comparable to that of aerogels. Based on the above conclusions, we provide a new strategy and correlated theories for preparing high-performance active–passive combining TPMs, which can simultaneously resist instantaneous thermal attack and prolonged high-temperature attack.

## 4. Materials and Methods

### 4.1. Materials

AO monohydrate (AR) was purchased from Shanghai Macklin Biochemical Technology Co., Ltd. (Shanghai, China). Natural graphite powder was obtained from Shanghai Aladdin Biochemical Technology Co., Ltd. (Shanghai, China). PR (THC-400) was supplied by Taihang Flame-Retardant Polymers Co., Ltd. (Shanxi, China). Concentrated sulfuric acid (98%), Hexamethylenetetramine (HTMA), L-ascorbic acid (AR), and absolute ethanol (AR) were procured from Sinopharm Chemical Reagent Co., Ltd. (Shanghai, China). Zirconia fibers (ZFs, YL-YZF-II) were provided by Nanjing Ligong Yulong New Material Technology Co., Ltd. (Nanjing, China).

### 4.2. Preparation of Superfine AO Powder by Anti-Solvent Crystallization

In this study, anti-solvent crystallization was used to adjust the morphology of AO crystals. Specifically, commercial AO was firstly dissolved in deionized water via stirring in a water bath at 80 °C. Next, absolute ethanol with different volume ratios to deionized water, 1:1, 3:1, 5:1 and 10:1, was added slowly into the AO aqueous solution as the anti-solvent. The mixture was cooled naturally to room temperature under continuous stirring. The precipitated AO powders were then collected by vacuum filtration and dried at 60 °C.

### 4.3. Preparation of GdAPs

GdAPs were prepared via an in situ reduction for graphene oxide (GO) during the anti-solvent crystallization process. The GO suspension was prepared from pristine graphite powder according to a modified Hummer’s method as reported in our previous paper [56,57]. Then GO was dispersed into deionized water and ultrasonicated for 2 h. Next, AO crystals were added and completely dissolved in GO solution by stirring under an 80 °C water bath. Ascorbic acid was introduced as a reducing agent, and the reaction proceeded for 4 h to reduce GO. Subsequently, absolute ethanol was added slowly to precipitate GdAPs, and the mixture was cooled to room temperature. Finally, the product was collected by vacuum filtration and dried it at 60 °C.

### 4.4. Preparation of GA/PR Precursor and Derived Pyrolytic Aerogel

**Figure 6:**
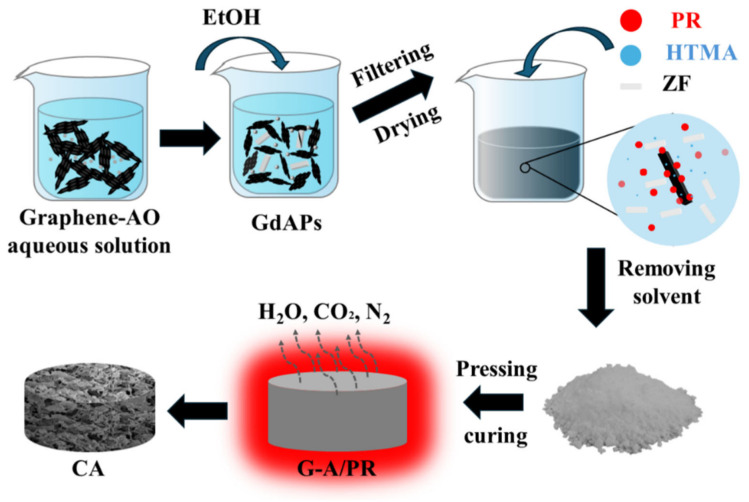
Schematic illustration of fabrication of GA/PR and CA.

The prepared GdAP powder was mixed with PR via a compression molding process to fabricate composite GA/PR materials. Firstly, a specified amount of PR and 20% HTMA by resin weight were added to ethanol and stirred until completely dissolved. GdAPs and ZFs were added to the resin solution and mechanically stirred to form a uniform slurry, ensuring the impregnation of the powder by the resin matrix. Subsequently, the resulting slurry was subjected to rotary evaporation at 80 °C to remove the solvent. The dried composite powders were transferred into a mold and then pressed into GA/PRs via the compression molding process. The obtained preforms were further cured in a 120 °C oven for 3 h. Finally, the GA/PR precursors were subjected to high-temperature pyrolysis to obtain the corresponding CAs. All the above preparation procedures are illustrated in Figure 6.

### 4.5. Characterization

The yield of precipitated AO powder under different anti-solvent ratios was measured as follows. Equal amounts of commercial AO were dissolved separately in identical volumes of deionized water. The anti-solvent of ethanol was then added at volume ratios ranging from 1:1 to 10:1 (ethanol : water) to induce precipitation. The resulting precipitate was collected by vacuum filtration and dried in a vacuum oven. The yield was calculated according to Equation (2):

(2) $$Yield\left(\%\right)=\frac{m_{1}}{m_{0}}\times 100\%$$

where *m*_0_ is the mass of the original AO, and *m*_1_ is the mass of the recrystallized AO. The crystal structure of AO was characterized using an X-ray diffractometer (XRD, Empyrean, Malvern Panalytical, Malvern, UK), which was recorded with a scanning rate of 1° per minute in a 2θ range from 5° to 80° with a Cu-target X-ray tube (Kα_1_ = 1.5405980 Å). The morphology of the AO crystals and CAs was characterized using a field-emission scanning electron microscope (SEM, JSM-7500F, JEOL, Tokyo, Japan). The thermogravimetric analysis (TGA) and differential scanning calorimetry (DSC) results of AOs and GA/PRs were evaluated using simultaneous thermal analysis (STA, Mettler Toledo, Greifensee, Switzerland). The measurement was conducted under a nitrogen atmosphere, with the sample heated from room temperature to 600 °C at a constant rate of 10 °C/min. The thermal conductivity of the GdAPs and CAs was determined using a thermal constant analyzer (TPS2500S, Hot Disk, Göteborg, Sweden) via the steady-state heat flow method. The compressive properties of the composite materials and the aerogels were evaluated using a universal testing machine (RGM-3005, Reger, Shenzhen, China). The specific surface area of the aerogel was determined by nitrogen adsorption–desorption measurements using an automatic surface area analyzer (ASAP 2460, Micromeritics, Norcross, GA, USA) with the Brunauer–Emmett–Teller (BET) method.

## Data Availability

The data presented in this study are openly available in the article.

## Supplementary Materials

The following supporting information can be downloaded at https://www.mdpi.com/article/10.3390/gels12060535/s1, Figure S1: SEM of AP-3. Figure S2: XRD patterns of AO powders prepared using different antisolvent content. Figure S3: TGA and DSC Curves of a Composite Material with 10% PR. Figure S4: Volume shrinkage of composite precursors with different resin contents. Figure S5. TGA and DSC curves of composite precursors with 5% ZFs. Figure S6: Photograph of commercial silica aerogel. Figure S7. Temperature curves of prepared CAs after multiple cycles. Figure S8. Obtained CA after pyrolysis at 1300 °C.

## Abbreviations

The following abbreviations are used in this manuscript:

AP	Ammonium oxalate powder
GdAP	Graphene-doped AO powder
GA/PR	GdAP and PR composite precursor
CA	Carbon aerogel
ZFs	Zirconia fibers
AO/PR	Ammonium oxalate and PR composite precursor
