# Supplementary material for: Graphene-Doped Ammonium Oxalate-Derived Carbon Aerogel with Controllable Structure for Synergistic Endothermic-Insulating Efficient Thermal Protection
Source: Gels. 2026 Jun 14;12(6):535. doi: 10.3390/gels12060535 (PMC13298003; doi:10.3390/gels12060535)
Supplement: Supplementary file 1 [file gels-12-00535-s001.zip › gels-4361354-supplementary.pdf]

Supporting Information

Graphene-doped Ammonium Oxalate-Derived Carbon Aerogel With Controllable Structure for Synergistic Endothermic-Insulating Efficient Thermal Protection

Zhengyang Lu, Guomin Ding *, Qilin Mei, Borui Zheng, Kun Chen, Hong Wang, Xu Han and Jiayang Shao

School of Materials Science and Engineering, Wuhan University of Technology, 122 Luoshi Road, Wuhan 430070, China
Correspondence: sdscdgm@126.com; Tel: +86 156-2383-5162

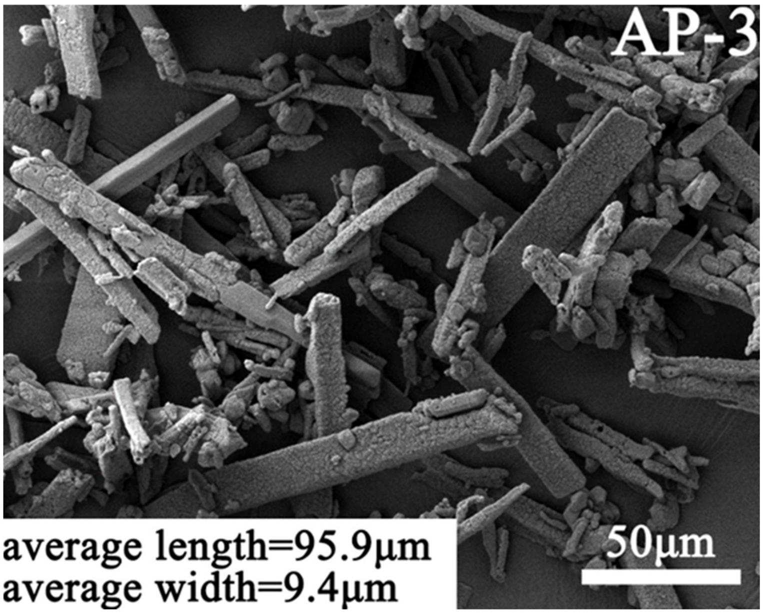

Figure S1. SEM of AP-3.

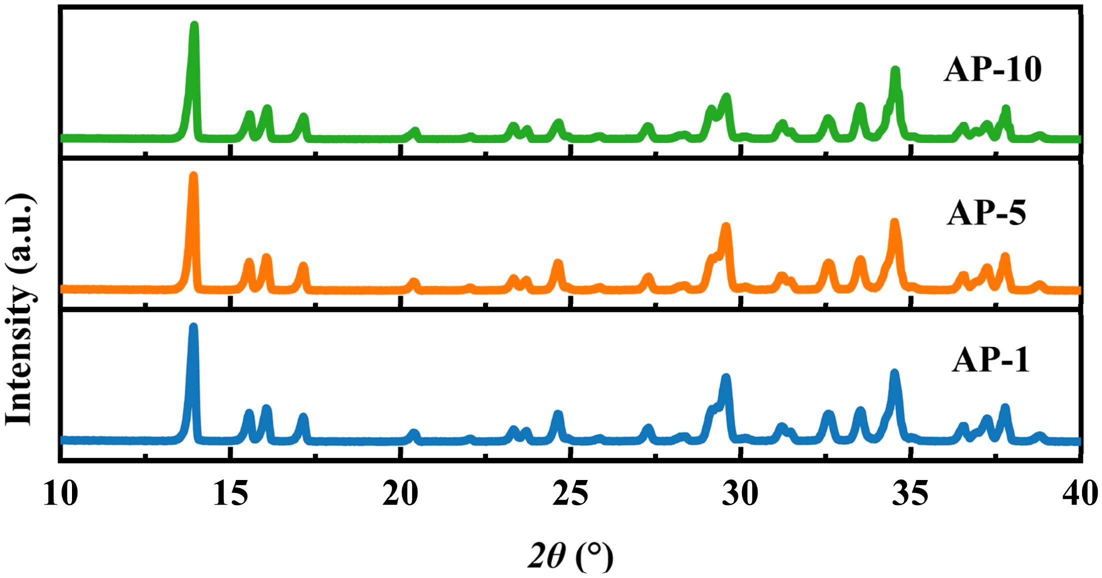

Figure S2. XRD patterns of AO powders prepared using different antisolvent contents.

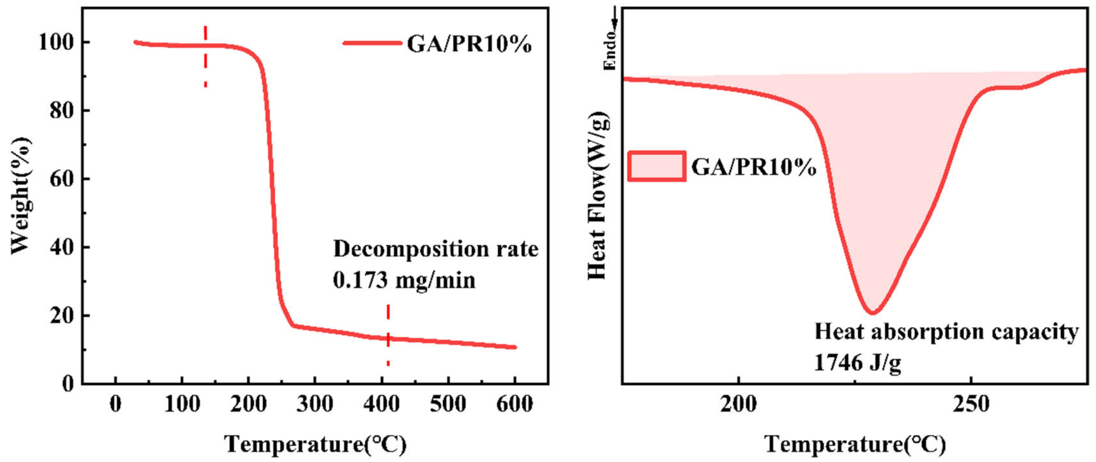

Figure S3. TGA and DSC curves of composite material with 10% PR.

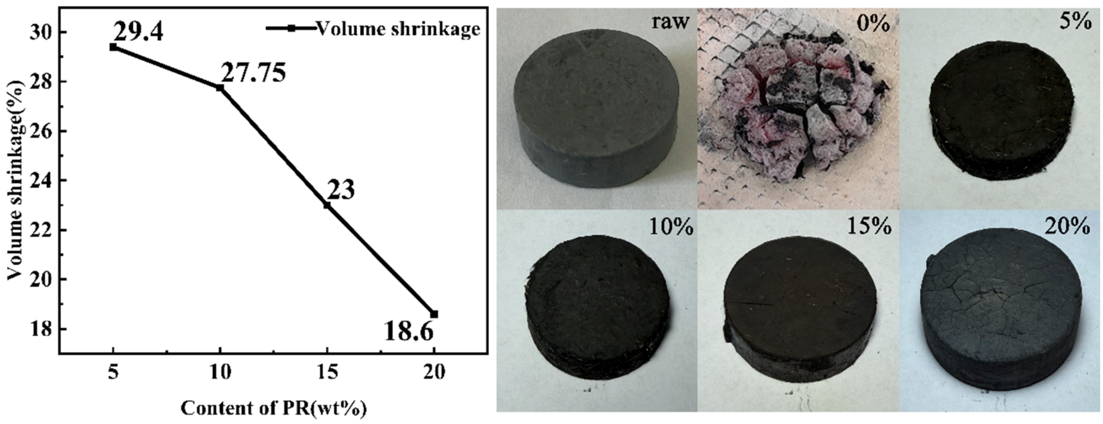

Figure S4. Volume shrinkage of composite precursors with different resin contents.

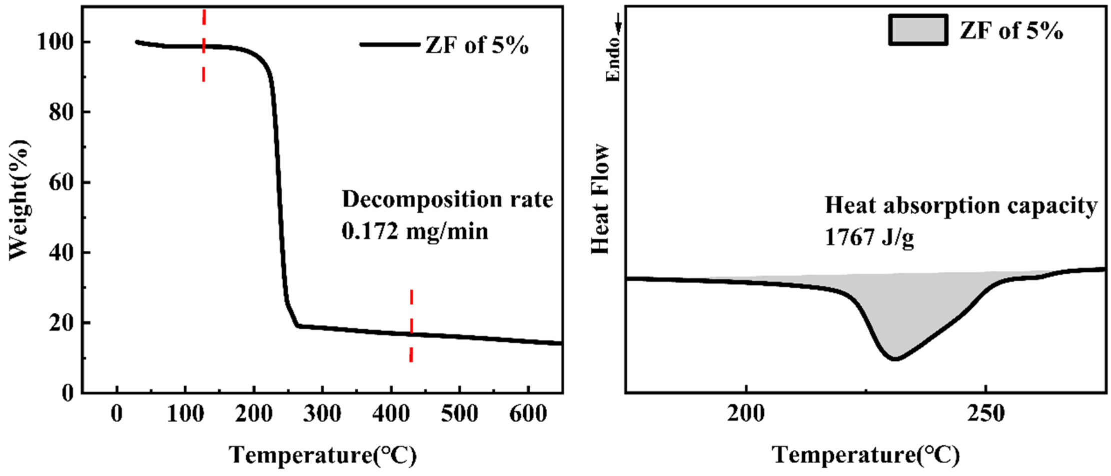

Figure S5. TGA and DSC curves of composite precursors with 5% ZFs.

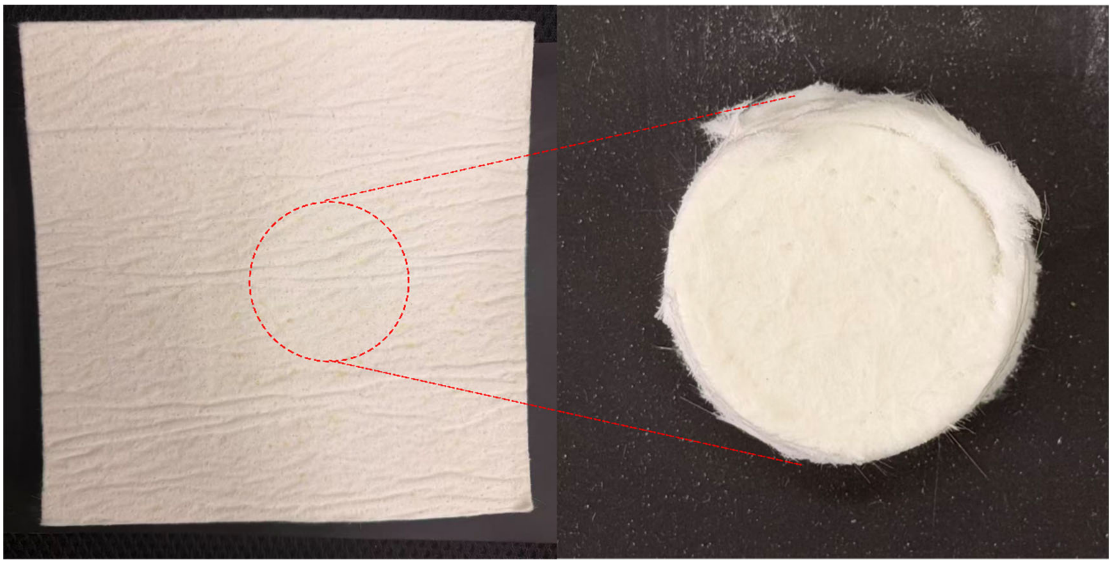

Figure S6. Photograph of commercial silica aerogel.

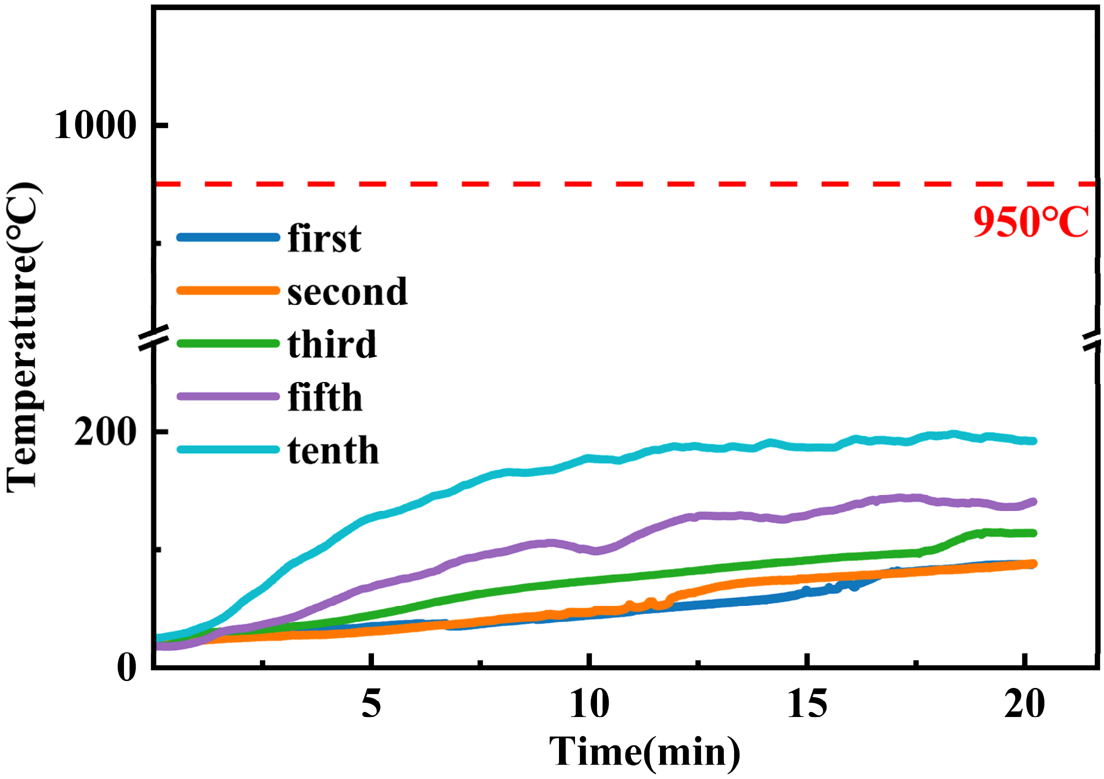

Figure S7. Temperature curves of prepared CAs after multiple cycles.

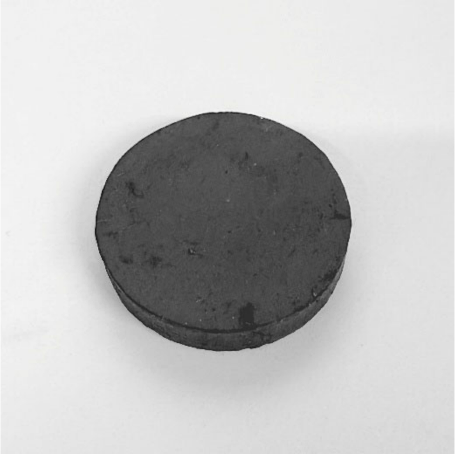
.

Figure S8. Obtained CA after pyrolysis at 1300 °C.
